# Is there a correlation between hs-CRP levels and functional outcome of Ischemic Stroke?

**DOI:** 10.12669/pjms.291.2799

**Published:** 2013

**Authors:** Aliakbar Taheraghdam, Siamak Aminnejad, Ali Pashapour, Reza Rikhtegar, Kamyar Ghabili

**Affiliations:** 1 Aliakbar Taheraghdam, MD, Neuroscience Research Center, Tabriz University of Medical Sciences, Tabriz, Iran.; 2 Siamak Aminnejad, MD, Students’ Research Committee, Tabriz University of Medical Sciences, Tabriz, Iran.; 3 Ali Pashapour, MD, Neuroscience Research Center, Tabriz University of Medical Sciences, Tabriz, Iran.; 4 Reza Rikhtegar, MD, Neuroscience Research Center, Tabriz University of Medical Sciences, Tabriz, Iran.; 5 Kamyar Ghabili, MD, Physical Medicine and Rehabilitation Research Center, Tabriz University of Medical Sciences, Tabriz, Iran.

**Keywords:** Atherosclerosis, C-reactive protein, Functional outcome, Inflammation, Ischemic stroke, Modified Rankin scale

## Abstract

***Objective:*** C-reactive protein, a well known marker of inflammation is being investigated as a probable marker of predicting acute cardiovascular events and its severity. The aim of the present study was to assess the possible role of highly-sensitivity C-reactive protein (hs-CRP) in predicting short-term functional outcome of ischemic stroke.

***Methodology:*** A prospective study was conducted on subjects admitted with first attack of confirmed ischemic stroke. It included 50 male and 52 female. Serum hs-CRP was measured in the 2^nd^ (CRP-D2) and 5^th^ days (CRP-D5) post-stroke. Modified Rankin scale (MRS) was measured in all subjects in the 2^nd^ (MRS-D2), 5^th^ days (MRS-D5) and also 3 month (MRS-M3) after stroke to assess the short-term functional outcome and mortality of subjects.

***Results:*** The mean age of the patients was 71.75±11.44 years. The mortality rate was 47.1% in the third months after stroke. There was no significant correlation between CRP-D2 and MRS-M3 and also between CRP-D5 and MRS-M3 (*P*>0.05). However there was a significant association between high CRP-D2 (CRP>3) and MRS-M3 and also between high CRP-D5 and MRS-M3 (*P*<0.005).

***Conclusion:*** This study showed that the value of CRP by itself could not predict the severity of short-term functional disability and it might not be useful as a clinical tool for predicting outcome.

## Introduction

 C-reactive protein (CRP) is an acute phase protein produced in response to inflammatory process and therefore it is regarded as a well-known marker of inflammation.^1^ CRP is currently being investigated as a probable marker of generalized atherosclerosis. Atherosclerosis is considered a chronic inflammatory response by arterial endothelium.^2^ Recently, the role of CRP as a reliable marker in predicting acute coronary syndrome and its outcome has been established.^3^

 There are evidences that inflammatory response is a part of ischemic stroke course^4^, therefore it might be hypothesized that a more severe stroke is associated with greater inflammatory response. Some studies have found evidence for CRP as a predictor of future stroke attack and its severity^5^^,^^6^, while others have not.^7^ Some studies have evaluated the role of post-ischemic CRP in long-term mortality of patients suffering from stroke.^8^^-^^10^ There has been only a few studies concerning the possible role of CRP as a predictor of functional outcome after stroke attack and there has been discrepancies regarding the results of these studies.^7^^,^^8^^,^^11^^-^^14^ The aim of the present study was to assess the possible role of highly sensitivity CRP (hs-CRP) in predicting functional outcome of patients admitted with ischemic stroke.

## Methodology

 This study was a prospective study conducted in Neuroscience Research Center, Tabriz University of Medical Sciences, Iran from August 2009 to August 2010. It included 50 male and 52 female patients. The study was approved by local ethics committee at Tabriz University of Medical Sciences. After explaining the study process in detail to the patients, written informed consent was obtained from all of the participants. Patients of at least 40-year-old, who were admitted with first-time ischemic brain stroke, were chosen sequentially. Stroke was defined as developing clinical evidence of focal neurologic deficits lasting more than 24 hours. Full neurologic physical examination was performed in all subjects and the diagnosis was confirmed with either computed tomography (CT) scan or magnetic resonance imaging (MRI). Subjects in whom physical examination or imaging modality was not consistent with the diagnosis of ischemic stroke were not included in this study. Patients were excluded if they had any history of acute coronary syndrome or infectious disease within last month.

 Furthermore, erythrocyte sedimentation rate (ESR) was measured in all subjects and those with abnormal ESR were not included in this study. Known risk factors of ischemic stroke were assessed and recorded in all patients. Serum total cholesterol concentration was measured while patients were under their regular regimens and subjects with hypercholesterolemia were identified. Blood pressure was measured in all subjects in a sitting position for three times and the average was taken as the reference. Subjects with a systolic blood pressure ≥140 mmHg or a diastolic blood pressure ≥90 mmHg were defined as having hypertension. Fasting blood glucose (FBS) and random glucose was measured in all subjects who were not under treatment with glucose-lowering agents and those with a repeated FBS≥126 mg/dL or a repeated random blood glucose ≥200 mg/dL were considered as having diabetes mellitus.

 Venous blood samples were obtained from all subjects two (CRP-D2) and five days (CRP-D5) after the beginning of the stroke attack. Within one hour of collection, the samples were centrifuged to separate the serum and were kept in -70°C. High-sensitivity CRP (hs-CRP) was measured using immunoturbidometric assays. CRP value of >3 was considered as high CRP.^15^

 Modified Rankin scale (MRS) was used to evaluate short-term functional outcome of stroke. MRS was conducted in all subjects two days (MRS-D2), 5 days (MRS-D5) and 3 months (MRS-M3) after the stroke attack. The patients were subject to MRS scores from 0 to 6 (0 = no symptoms, 1 = no significant disability, 2 = slight disability, 3 = moderate disability, 4 = moderately severe disability, 5 = severe disability, and 6 = dead).

 Data were presented as mean ± standard deviation (SD). The statistical analysis was performed using SPSS for windows version 16.0 using Chi-square test and Mann-Whitney U test, whenever appropriate. A *P* value <0.05 was considered statistically significant.

## Results

 The mean age of the patients was 71.75±11.44 years (40-91 years). Only three patients died on the fifth day after stroke; however the mortality rate was 47.1% in the third months after stroke. The mean hs-CRP level at 2^nd^ and 5^th^ days was 11.61±9.45 mg/L (0.30-42) and 14.69±11.87 mg/L (1.1-49), respectively. There was no significant difference between the mean hs-CRP levels of day 2 and day 5 (*P*>0.05). Patients’ demographic data, hs-CRP levels and MRS scores are shown in Table-I.

**Table-I:** Patients’ Demographic Data, hs-CRP Levels and MRS Scores

*Variable*	*Total (n=102)*
Gender (male:female)	50:52
Age (years)	71.75±11.44
hs-CRP (day 2)	11.61±9.45
hs-CRP (day 5)	14.69±11.87
High hs-CRP (day 2)	88 (86.27%)
High hs-CRP (day 2)	95 (93.14%)
MRS score (day 2)	3.83±0.72
MRS score (day 5)	3.92±1.06
MRS score (month 3)	4.17±1.97
Hypertension, n (%)	51 (50%)
Diabetes mellitus, n (%)	16 (15.7%)
Hyperlipidemia, n (%)	9 (8.8%)

 With regard to the MRS scores, MRS-M3 significantly correlated with MRS-D2 (r=0.44, *P*<0.005) and MRS-D5 (r=0.74, *P*<0.005). Furthermore, there was no significant correlation between CRP-D2 and MRS-M3 (*P*=0.85) and also between CRP-D5 and MRS-M3 (*P*=0.59). However there was a significant association between high CRP-D2 (CRP>3) and MRS-M3 (*P*<0.005, Table-II) and also between high CRP-D5 and MRS-M3 (*P*<0.005, Table-II). Furthermore, there were no significant differences between hypertension, diabetes mellitus, hyperlipidemia and hs-CRP levels and MRS scores at the studied times (*P*>0.05).

**Table-II:** MRS Score at 3 Months between Patients with Low and High CRP Levels

		*MRS-M3*			*P value*
		*0-1*	*2*	*3-6*	
CRP-D2	Low (<3)	6	0	8	<0.005
	High (>3)	5	12	71	
CRP-D5	Low (<3)	2	1	4	<0.005
	High (>3)	9	11	75	

## Discussion

 The present study did not find any correlation between hs-CRP levels and short-term (three months) functional outcome of ischemic stroke. Similar to the present study, Canova et al^8^ and Modrego et al^12^ failed to conclude any relationship between CRP and outcome of acute cerebrovascular events such as ischemic stroke. In contrast, CRP levels have been correlated positively with the size of the infarct and stroke severity.^11^^,^^16^ Furthermore CRP elevation in ischemic stroke indicated a worse prognosis, as it has been associated with higher in-hospital mortality^17^^,^^18^ higher mortality at six months^19^, and more disability.^20^

 The present study also revealed that high CRP (CRP>3) was associated with poor short-term (three months) functional outcome of ischemic stroke. Similarly, Idicula and colleagues found a crude association between on admission high CRP and short-term (7 days) functional outcome in patients with acute ischemic stroke. They used Barthel index and MRS to evaluate the stroke outcome.^7^ Likewise, in a recent Korean study, Song et al^13^ demonstrated that elevated hs-CRP levels on the seventh hospital day, rather than within 24 h after stroke onset, could strongly predict the prognosis of functional disability, assessed by MRS score, 12 months after stroke onset. Moreover, elevated CRP levels have been associated with poorer one-month functional outcome, evaluated with Barthel index, in Malaysian patients with acute ischemic stroke.^21^ As CRP is produced as a response to brain tissue necrosis following ischemic stroke, it has been suggested as a valuable predictor of functional outcome.^11^

 The clinical importance of possible relation between CRP and functional outcome of stroke is unclear. At present, it is believed that there is not enough evidence to recommend measurement of CRP in the usual evaluation of cerebrovascular disease risk in primary prevention. Nonetheless, in secondary prevention of stroke, elevated CRP adds to current prognostic markers, although it remains to be established whether specific therapeutic options can be derived from this.^22^

 This study has several limitations. The prevalence of future vascular events was not assessed in this study and might be subject of other studies. Moreover, all the risk factors associated with ischemic stroke such as cigarette smoking and etc has not been studied in the present investigation. In addition, other functional outcome measurement scales such as Barthel index could have been applied in the present study. On the other hand, an advantage of the current study might be highlighted. Most of the studies evaluating the probable association of CRP and stroke outcome have based their results only on a sole measurement of CRP. CRP values might be affected by stress, infection or technical errors and therefore a once-measured high level cannot be trusted.^7^ CRP level was measured twice in this study to avoid the error of overestimation.

## Conclusions

 In conclusion, this study showed that the value of CRP by itself could not predict the severity of short-term functional disability and it might not be useful as a clinical tool for predicting outcome. 
